# High Expression of Interleukin-2 Receptor Subunit Gamma Reveals Poor Prognosis in Human Gastric Cancer

**DOI:** 10.1155/2021/6670834

**Published:** 2021-01-21

**Authors:** De-Ping Wang, Rong Zhao, Yue-Hong Qi, Jing Shen, Jia-Yi Hou, Mei-Yue Wang, Xiao-Gang Bi, Xiao-Qing Guo, Ji-Min Cao

**Affiliations:** ^1^Key Laboratory of Cellular Physiology at Shanxi Medical University, Ministry of Education, and the Department of Physiology, Shanxi Medical University, Taiyuan, China; ^2^Department of Anesthesiology, Shanxi Provincial People's Hospital, Taiyuan, China; ^3^Department of General Surgery, Shanxi Provincial People's Hospital, Taiyuan, China; ^4^Department of Liver Disease, Taiyuan Third People's Hospital, Taiyuan, China

## Abstract

Precision medicine for gastric cancer (GC) is still an unsolved issue, because most available target drugs are not specifically designed for GC. Exploring novel signaling molecules with target value for GC is in urgent need. This study aimed to reveal that interleukin-2 receptor subunit gamma (IL2RG) is such a key molecule in human GC progression. GC tissues and paracancerous gastric tissues were collected from 7 patients (5 males and 2 females) during tumor radical excision surgery. These tissues were used to identify the differentially expressed genes (DEGs) with RNA-seq and serial bioinformatics analyses including Kyoto Encyclopedia of Genes and Genomes (KEGG) pathway analysis, gene expression profiling interactive analysis (GEPIA), and survival analysis. RT-qPCR and western blotting were performed to compare the mRNA and protein expression levels of IL2RG between GC tissues and adjacent normal gastric tissues as well as between GC cell line SGC-7901 and normal gastric epithelial cell line GES-1. Results showed striking elevations of IL2RG both in the mRNA and protein levels in GC tissues and human gastric cancer SGC-7901 cell line compared, respectively, with the adjacent normal gastric tissues and normal GES-1 cells, and higher IL2RG expression was associated with lower survival. Analyses on the GSE29272 and GSE15459 datasets from Gene Expression Omnibus verified that IL2RG was highly expressed in GC patients and was associated with poor overall survival. In addition, molecular docking showed that a small molecule, resatorvid (TAK 242), might be an inhibitor of IL2RG. We conclude that IL2RG is overexpressed in advanced GC and is associated with low survival. IL2RG may serve as a biomarker of GC progression and poor prognosis.

## 1. Introduction

Gastric cancer (GC) has a mortality rate of the third place and a 5-year survival rate of less than 30% [1]. The high incidence of GC in the world is mainly reported in East Asia. Although current clinical diagnosis and treatment have been developing, GC patients are often diagnosed in the late stage, while metastasis and recurrence may occur after radical excision in the early stage. The pathogenesis of GC is not fully clear [2, 3], and this situation leads to difficulties in early diagnosis, treatment, and prognosis. Therefore, the long-term survival rate of GC patients is not optimistic. The recognized risk factors of GC include diet, smoking, family history, and helicobacter pylori infection.

At present, precision medicine for GC is still preliminary, as most of the available targeted drugs are not designed specifically for GC. Human epidermal growth factor receptor-2 (HER2), the first identified protein associated with breast cancer, is found overexpressed in GC [4]. Trastuzumab, a humanized monoclonal antibody designed for HER2, has been used in the therapy metastatic GC, but its safety evaluation is still lacking. Unlike treatment of breast cancer, trastuzumab used in GC does not appear to be effective after the first-line treatment failed. Moreover, trastuzumab causes adverse reactions, such as diarrhea, oral inflammation, anemia, thrombocytopenia, and fatigue [5]. Bevacizumab acts on endothelial growth factor (VEGF) and can improve the median progression-free survival of GC patients in advanced stage, but it is not effective in improving the median overall survival [6]. The therapeutic effect of bevacizumab is inferior to that of chemotherapy alone [7]. These studies suggest that HER2 and VEGF are not satisfactory drug targets for GC. Therefore, it is necessary to further understand the pathological signaling of GC and find novel GC specific molecular targets.

High-throughput sequencing has been widely used to detect the differences in transcriptome between tumor and normal tissues. Here, we performed a transcriptome study to compare the DEGs between GC tissues and paracancerous normal gastric tissues. KEGG pathway enrichment and GEPIA of the DEGs were performed to screen the pathways and the potential target genes. We found that IL2RG was prominent in human progressive GC, as verified by transcriptome profiles, RT-qPCR, and western blotting. The study provides a novel insight into the pathogenesis of GC and suggests that IL2RG may potentially serve as a marker or drug target for GC precision medicine.

## 2. Materials and Methods

### 2.1. Patients and Gastric Tissue Sampling

The study included seven inpatients (5 males and 2 females) diagnosed with gastric cancer (GC) in Shanxi Provincial People's Hospital, Taiyuan, China. Patient information is shown in Table S1. All the patients were in advanced stage and underwent radical tumor excision surgery. The GC tissues and paired adjacent normal gastric tissues were harvested during surgeries. These tissues were divided into six subgroups: male tumor (TM), female tumor (TF), mixed (male plus female) tumor (T), female normal (NF), male normal (NM), and mixed normal (N) groups. The harvested tissues were frozen in liquid nitrogen and stored at an ultra-low-temperature freezer for the following experiments. In addition, we obtained GSE29272 [8] and GSE15459 [9] datasets from Gene Expression Omnibus (GEO, https://www.ncbi.nlm.nih.gov/geo/). The mRNA expression profiles of GSE29272, which consists of 134 gastric cancer samples and paired paracancerous samples, were detected by Affymetrix Human Genome U133A Array. The mRNA expression profiles of GSE15459, which consists of 200 gastric cancer samples with complete survival information, were detected by Affymetrix Human Genome U133 Plus 2.0 Array.

### 2.2. RNA Isolation

Frozen gastric tissues were washed with precold phosphate buffer solution (PBS) to remove residual blood. Total RNA was extracted using RNAiso Plus reagent (TaKaRa, Osaka, Japan). RNA concentrations were quantified using NanoDrop (Specialized equipment, Thermo Fisher Scientific, Waltham, USA). The quality of the total RNA was tested using 1% (w/v) agarose gel electrophoresis.

### 2.3. cDNA Library Preparation and RNA Sequencing

One *μ*g RNA per sample was used in a volume of the 25 *μ*l reverse transcriptional system. The cDNA library was built with the PrimeScript™RT reagent kit (TaKaRa, Osaka, Japan) using PCR amplification. RT-qPCR reaction reagent was prepared using TB Green™Premix Ex Taq™II (TaKaRa, Osaka, Japan). The library quality was tested with Agilent 2100 Bioanalyzer (Agilent Technologies Inc., CA, USA). After cluster generation, the library was sequenced by Illumina HiSeq 4000 platform (Illumina, San Diego, USA). The quality control of the sequencing data is shown in Table S2. The numbers of bases in all samples were between 6.7 G and 7.3 G. No guanosine-cytidine (G-C) bias was found. The data set quality met the requirement for analysis with mapping rates of all samples >96%.

### 2.4. Analysis of RNA-Seq Data

The sequencing reads with inferior quality or adaptors were filtered. Trimmed reads were mapped using HISAT2 software [10] to the reference human genome (hg38) and then were processed with StringTie software [11] for transcript splicing analysis. The differentially expressed genes (DEGs) were analyzed by DESeq2 [12] with |logFC| > 1 (FC, fold change) and FDR-adjusted *p* value <0.01 as thresholds.

### 2.5. Function Enrichment Analysis

ClusterProfiler R package [13, 14] was used to perform KEGG pathway enrichment analysis, and Kaplan–Meier survival analysis was conducted using the GEPIA database. *p* < 0.05 denoted the significance of enriched pathways for the DEGs.

### 2.6. Cell Lines and Culture

The SGC-7901 human GC cell line and GES-1 human gastric epithelial cell line were purchased from Shanghai Institutes for Biological Sciences, Chinese Academy of Sciences (Shanghai, China). The SGC-7901 cells were cultured in a Roswell Park Memorial Institute (RPMI) 1640 culture medium, and GES-1 cells were cultured in Dulbecco's modified Eagle's medium (DMEM) (Hyclone, South Logan, UT, USA) containing 10% fetal bovine serum (FBS) (Gibco, NY, USA) at 37°C in a humidified incubator with 5% CO_2_. Cells cultured to about 80% confluency were used for in vitro assays.

### 2.7. RT-qPCR

RT-qPCR was performed to examine the levels of IL2RG mRNA both in gastric cell lines and gastric tissues. Total RNAs of tissues and cells were extracted using RNAiso Plus reagent (TaKaRa, Osaka, Japan). The concentration and quality of RNA were examined by Nanodrop. PrimeScript™ RT reagent Kit (TaKaRa, Osaka, Japan) was used to reversely transcript the RNA into cDNA. RT-qPCR was performed according to the instructions of TaKaRa TB Green™ Premix Ex Taq™ II, with GAPDH as the internal reference. The mRNA levels of IL2RG were calculated using 2 − ∆∆Ct method. The primer sequences for IL2RG were as follows: forward, GTTCTCCTTGCCTAGTGTGGATGG; reverse, CCAACAGAGATAACCACGGCTTCC. The primer sequences for GAPDH were as follows: forward, CAAGGTCATCCATGACAACTTTG; reverse, GTCCACCACCCTGTTGCTGTAG.

### 2.8. Western Blotting

Proteins used for western blotting were extracted from GC tissues, adjacent normal gastric tissues of each subgroup, SGC-7901 cells, and GES-1 cells. Proteins were quantified with bicinchoninic acid (BCA) assay. A total amount of 40 *μ*g protein of each sample were separated by 10% SDS-PAGE and then transferred to PVDF membranes. After blocking with PBS containing 5% nonfat dry milk for one hour, the membranes were incubated with mouse anti-IL2RG (dilution 1 : 400) (Abcam, Cambridge, UK) and rabbit anti-GAPDH (1 : 1500) (ZSGB-Bio, China) at 4°C for overnight. Then, the membranes were incubated with horseradish peroxidase-labeled secondary anti-mouse (1 : 10000) (ZSGB-Bio, Beijing, China) or anti-rabbit (1 : 10000) (ZSGB-Bio, China) antibodies for one hour at room temperature after washing with PBST. Target immunoreactive bands were visualized with enhanced chemiluminescent substrate (Boster Biological Technology, Wuhan, China). Densitometric analysis was performed with ImageJ software to evaluate the target proteins levels. GAPDH was used for normalization.

### 2.9. Molecular Docking

The initial structure for IL2RG was extracted from the structure of interleukin-2 variant in complex with interleukin-2 receptor (PDB ID: 5M5E). The docking poses were determined by AutoDock-1.5.6. Result showed that resatorvid could dock with IL2RG. The top 10 poses were picked up based on the calculated binding affinities using the scoring function in the AutoDock. The best binding poses of IL2RG and resatorvid were used to determine the interaction between IL2RG and resatorvid. The binding mechanisms of resatorvid to IL2RG were characterized using LigPlot, and the image of the model was generated using PyMOL software.

### 2.10. Statistical Analysis

Analysis of variance and Student's *t*-test were used for comparisons of gene expression levels among groups. GEPIA [15] based on the Cancer Genome Atlas (TCGA) database and KMplotter (http://kmplot.com/analysis) were used to evaluate the overall and relapse-free survival of GC, and the differences between the two groups were tested by log-rank. All statistical analyses were calculated in R software (version 3.4.1), and *p* < 0.05 denoted the statistical significance.

## 3. Results

### 3.1. Identification of DEGs

**Figure 1:**
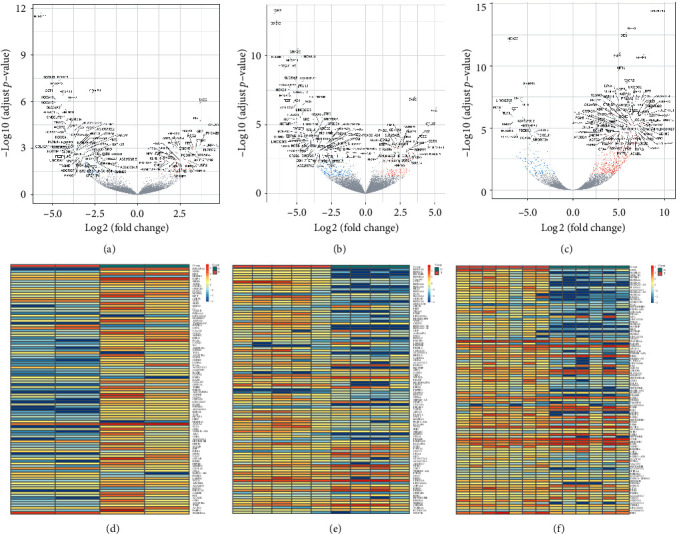
The DEG identification in different gastric tissues. (a)–(c) Volcano plots of DEGs, respectively, for female GC tissues (a), male GC tissues (b), and mixed GC tissues (c). The abscissae indicate the fold change of DEGs in each subgroup of tissues, and the ordinates represent the statistical significance of the DEGs. Blue dots indicate the downregulated genes, and red dots indicate the upregulated genes. (d)–(f) Heatmaps of DEGs, respectively, for female GC tissues (d), male GC tissues (e), and mixed GC tissues (f). Lines represent different genes, and columns represent samples. Red: upregulated expression. Blue: downregulated expression.

All sequencing samples were compared on the expression level of transcripts per million (TPM), and the distribution of expression level among all samples was consistent (Figure S1), which met the requirement for DEG analysis. Figures 1(a)–1(c) show the volcano plots of the top 100 annotated DEGs, respectively, in female, male, and mixed GC tissues. Compared with respective normal gastric tissues, female GC showed 1057 DEGs (Figure 1(a)), male GC showed 270 DEGs (Figure 1(b)), and the mixed GC exhibited 710 DEGs (Figure 1(c)). Figures 1(d)–1(f) show the heatmaps exhibiting the expression levels of the DEGs, respectively, for female (Figure 1(d)), male (Figure 1(e)), and mixed (Figure 1(f)) GC and non-GC gastric tissues. Each column denotes a tissue subtype.

### 3.2. Comparison of DEG and KEGG Enrichments

**Figure 2:**
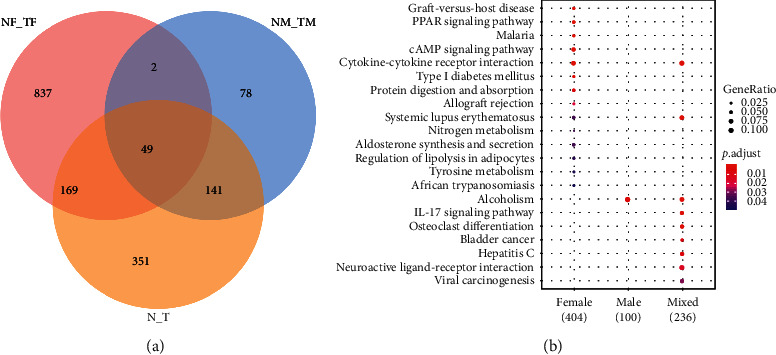
Comparison of DEG and KEGG enrichment analysis. (a) Venn diagrams revealing uniquely and commonly expressed genes. NF, female adjacent normal gastric tissue. TF, female tumor tissues. NM, male adjacent normal gastric tissues. TM, male tumor tissues. N, mixed (female plus male) adjacent normal gastric tissues. T, mixed tumor tissues. A total of 2037 genes exhibited significantly (*p* < 0.01) different levels of expression across three (female, male, and mixed) tumor groups compared to the respective normal tissue groups. Numbers in the figure indicate the DEG numbers that were uniquely and commonly expressed in each group. (b) KEGG enrichment analysis of DEGs in female, male, and mixed tumor groups. Dot size and color indicate gene ratio and *p* value, respectively.

To demonstrate the common and unique genes among tissue subgroups, cross-comparison of DEGs was performed for female, male, and mixed subgroups.  Figure 2(a) shows that the numbers of DEGs in the female group were higher than that in other two groups (male and mixed), while the male group shows more consistency with the mixed group. There were 49 overlapping DEGs among the three groups.

In order to improve the accuracy of the results, the regulation information of TF-target and miRNA-target, which had been verified by experiments, was collected (Figure S3). TF-target and miRNA-target data in human genome were derived from TRRUST (Version 2) and miRTarBase (version 8.0), respectively. Only the miRNA-target relationship with strong data support was extracted from the miRTarBase (version 8.0) and was used for further analysis. The TF-target regulation information of human, total of 8427 pairs, was extracted from the TRRUST (version 2) database, which involved 795 TFs and 2492 target genes. The miRNA-target information with strong data support was extracted, 8157 pairs in total, which involved 735 miRNAs and 2767 target genes.

Compared with the NF group, there were a total of 1057 DEGs in the TF group, of which 250 were upregulated and 807 were downregulated. Compared with the NM group, there were a total of 270 DEGs in the TM group, of which 142 were upregulated and 128 were downregulated. Compared with the N group, there were 710 DEGs in the T group, of which 403 were upregulated and 307 were downregulated (Figure S3). The intersection of three groups was extracted, and there were a total of 49 DEGs, of which 35 were upregulated and 14 were downregulated. Based on the 8427 pairs of TF-target regulation information in the TRRUST (Version 2) database, searching DEG-regulating TF, 44 pairs of TF-DEG regulation relationships were constructed, involving 14 DEGs and 38 TFs. Based on the 8157 pairs of miRNA regulation information in the miRTarBase (version 8.0) database, searching DEG-regulating miRNA, 46 pairs of miRNA-DEG regulation relationships were constructed, involving 14 DEGs and 40 miRNAs (Figure S3).

The biological processes associated with DEGs were determined by the KEGG pathway enrichment analysis.  Figure 2(b) shows the DEG enrichment in female GC, male GC, and mixed GC tissues. Female GC mainly manifested with enrichments of cytokine-cytokine receptor interaction (CCRI) and systemic lupus erythematosus, etc. Male GC mainly exhibited enrichment of the alcoholism pathway. Mixed GC showed enrichments of multiple pathways including CCRI, systemic lupus erythematosus, and alcoholism.

### 3.3. Association of IL2RG Upregulation with GC Progression and Prognosis

Since CCRI was enriched in both the female tumor group and mixed tumor group (Figure 2(b)), it may play a vital role in GC progression. Based on the DEGs identified in the GC and non-GC tissues, KEGG pathway enrichment analysis shows that IL2RG was enriched in the CCRI pathways and was upregulated in GC (Figure S2).

**Figure 3:**
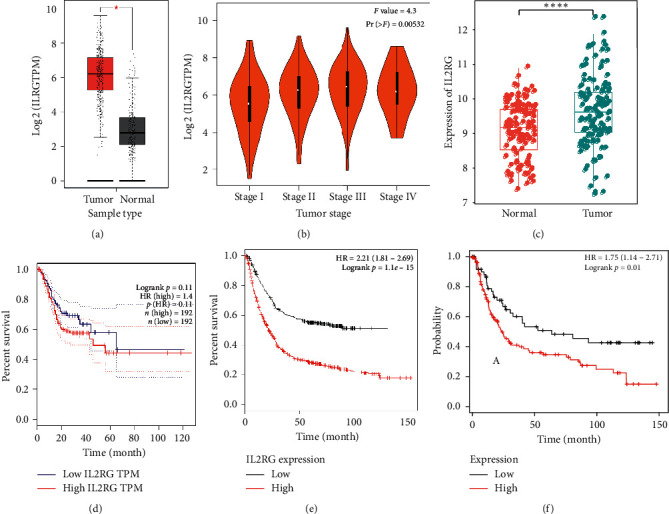
IL2RG expression level and survival analysis. (a) IL2RG expression levels in tumor tissues and normal tissues (stomach adenocarcinoma, STAD). IL2RG gene expression level was significantly elevated in tumor tissues T (*n* = 408) compared to the paracancerous tissues N (*n* = 211). ^*∗*^*p*=1.1*e* − 15. (b) The correlation of the expression level of IL2RG and the cancer pathologic stage (TCGA data analysis in GEPIA). (c) IL2RG expression levels in normal and tumor samples in the GSE29272 dataset. ^*∗∗∗∗*^*p* < 0.001. (d) Relapse-free survival analysis of IL2RG in gastric cancer based on TCGA data analysis in GEPIA. HR: hazard ratio. (e) Kaplan–Meier curves showed a strong correlation of the IL2RG expression and overall survival rate. TPM, transcripts per million. (f) Overall survival curve of patients with high and low expression levels of IL2RG in the GSE15459 dataset.

To further verify the importance of IL2RG in the CCRI pathways, we used GEPIA to explore the expression level of IL2RG in the massive data of GC. IL2RG in GC was overexpressed (*p* < 0.001) compared with the adjacent gastric tissue (Figure 3(a)). The IL2RG level was gradually higher with the prolongation of GC pathological stages (Figure 3(b)). Similarly, IL2RG was found highly expressed in gastric cancer samples compared with normal samples in the GSE29272 dataset (Figure 3(c)). Subsequently, the relationship between IL2RG and relapse-free survival as well as overall survival was investigated using GEPIA and KMplotter, respectively. Although there was no difference (*p*=0.11) between the two groups with high and low expression of IL2RG in relapse-free survival, the relapse-free survival of the group with higher expression of IL2RG was lower than the group with low expression of IL2RG (Figure 3(d)), while higher expression of IL2RG was closely related to poor overall survival in GC (*p*=1.1*e* − 15) (Figure 3(e)). It was also found that patients with higher expression level of IL2RG had poor overall survival in the GSE15459 dataset (Figure 3(f)).

### 3.4. Validation of IL2RG Abundance in GC Tissue and GC Cell Line

**Figure 4:**
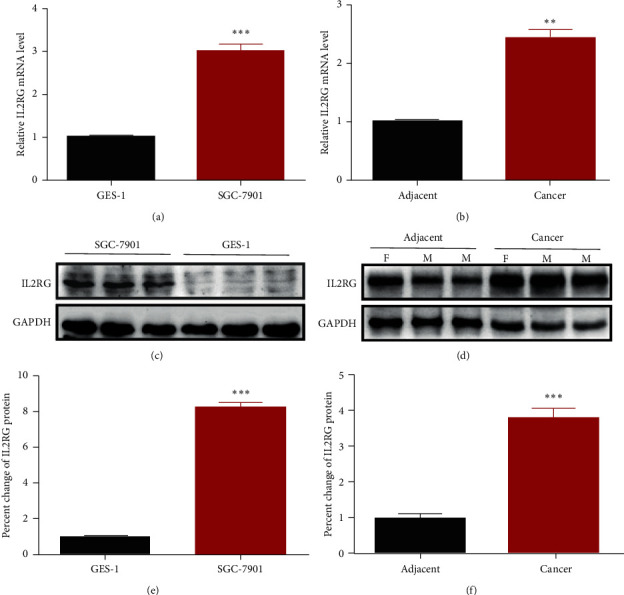
Western blotting and RT-qPCR showing the IL2RG expression levels in gastric tissues and gastric cell line. (a), (b) mRNA levels of IL2RG in SGC-7901 cells and GES-1 cells and cancer tissues and paracancerous tissues, respectively. (c), (d) Representative electrophoresis bands of western blots for IL2RG protein expression in SGC-7901 cells and GES-1 cells, as well as in cancer tissues and paracancerous tissues, respectively. GAPDH was loading control. (e), (f) statistical protein levels of IL2RG in SGC-7901 cells and GES-1 cells, as well as in GC tissues and paracancerous tissues. ^*∗∗*^*p* < 0.01,^*∗∗∗*^*p* < 0.001 vs. GES-1 cells or adjacent normal gastric tissues.

The mRNA levels of IL2RG were much higher both in SGC-7901 cells (Figure 4(a)) and in GC tissues (Figure 4(b)) compared, respectively, with GES-1 cells and paracancerous gastric tissues as verified with real-time qPCR analysis. Consistent with the mRNA levels, western blotting results show that IL2RG expression levels were also significantly elevated both in SGC-7901 cells (Figures 4(c) and 4(e)) and GC tissues compared with GES-1 cells and paracancerous tissues (Figures 4(d) and 4(f)).

## 4. Discussion

GC is one of the most prevalent and lethal cancers in the world especially in Asia, and the therapy is still disappointing. In order to explore new target genes and pathological mechanisms of GC, we performed a high-throughput sequencing of mRNAs extracted from GC patients. We reported the DEGs in the male GC and the female GC tissues relatively to the adjacent normal gastric tissues and analyzed the functions of these DEGs using KEGG enrichment analysis and identified the potential key gene involved in the enriched pathways.

This study focused on the role of IL2RG in GC which has been seldom reported. Comparing with the normal gastric tissues from females, the DEGs associated with CCRI were enriched in female GC tissues. It is worth noting that IL2RG, which is involved in the CCRI pathways, was enriched in GC (Figure S2). IL2RG is a shared signaling subunit of several interleukin receptors involved in the immune system, including IL-2, IL-4, IL-7, IL-9, IL-15, and IL-21, thus referred to as the common gamma chain (*γ*c) [16]. *γ*c is vital for the formation and homeostasis of T cells and natural killer cells. It has been reported that *γ*c is involved in tumorigenesis and affects malignant cell differentiation, activation, and proliferation by influencing tumor environment. *γ*c may promote malignant transformation of cells or conversely lead to tumor regression [17, 18]. In addition, *γ*c-induced JAK-STAT signaling pathways regulate immune response and have been related to some cancers [19]. We showed here that IL2RG was upregulated in GC tissues and high IL2RG predicted poor overall survival, suggesting that IL2RG may play a carcinogenic role in the development of human GC. We also verified our findings in datasets from the GEO database and confirmed that IL2RG is overexpressed in patients with gastric cancer and is associated with poor survival.

A question may come up whether IL2RG is a proto-oncogene for GC. It is known that proto-oncogenes are some normal endogenous genes which regulate cell proliferation, differentiation, and some other cellular activities at physiological conditions. Abnormal activation of the proto-oncogene may become carcinogenic. Ways of proto-oncogene activation include cell obtaining promoter and enhancer from retrovirus, gene translocation, amplification, or point mutation. This study demonstrated high expression of IL2RG in GC tissues and GC cell line both in the transcriptional level and the translational level, suggesting that IL2RG may potentially be a proto-oncogene in GC which promotes GC progression via the way of amplification. This possibility warrants further verification by experiments such as IL2RG gene sequencing or silencing.

Using molecular docking software, we found a small molecule named resatorvid or TAK 242, which was identified by Takeda Pharmaceuticals and was developed to inhibit inflammatory mediators' production [20]. A study showed that blockade of the TLR4 receptor with resatorvid could be a promising approach to enhance anticancer effects of chemotherapy [21]. We showed here that the Asn44 of IL2RG was an important site to interact with resatorvid (Figure S4), suggesting that resatorvid may be an inhibitor of IL2RG. The molecular docking approach might be a way to find IL2RG inhibitors, which may have therapeutic potential for GC.

In addition to IL2RG, the study also revealed the role of alcoholism in GC pathogenesis and progression which has been debated for a long time as reflected by inconsistent investigation results [22–24]. Here, we provide bioinformatic evidence derived from human GC tissues that alcoholism is an important risk factor and facilitates the development of GC, especially in men.

In summary, we demonstrated DEGs in the male, female, and mixed GC tissues relatively to respective adjacent normal gastric tissues. We further identified IL2RG overexpression in human GC tissues and GC cell line and its positive association with poor prognosis. These findings are seldom reported before. We suggest that IL2RG may serve as a biomarker or a potential drug target for GC. Pathway enrichment analysis allows a better understanding on GC pathology and may help to find rational target gene for GC therapies.

## Data Availability

The datasets of this study are available on request to the corresponding author.

## Additional Points

A major limitation of the present study was the limited study population due to difficulty in getting GC samples during the COVID-19 occasion. However, the focus of this study was not based on large-scale population experiments but concentrated on determination of IL2RG as a potential biomarker of GC. Analyses on three databases (GEPIA, GSE29272, and GSE15459) and experiments of GC cell line may support the conclusion of the study. Expanded population studies are warranted in the future to further determine the role of IL2RG in GC.

## Supplementary Materials

Supplementary Materials Table S1: characteristics of the patients diagnosed with gastric cancer. Table S2: information of raw sequencing reads and mapping. Figure S1: sequencing samples comparison based on the expression levels of transcripts per million (TPM). N, normal gastric tissues. T, tumor. F, female. M, male. Figure S2: cytokine-cytokine receptor interaction (CCRI) pathway map based on the DEGs between GC and adjacent non-GC gastric tissues. Figure S3: the regulation network of TF-DEG and miRNA-DEG.A. Figure S4: molecular docking results of resatorvid and IL2RG. A. The files named deg_N_T_tpm.txt, deg_NF_TF_tpm.txt, and deg_NM_TM_tpm.txt contain the DEGs and related statistical information.

## References

[1] Goetze O. T., Al-Batran S.-E., Chevallay M., Mönig S. P. (2018). Multimodal treatment in locally advanced gastric cancer. *Updates in Surgery*.

[2] Baroudi O., Benammar-Elgaaied A. (2016). Involvement of genetic factors and lifestyle on the occurrence of colorectal and gastric cancer. *Critical Reviews in Oncology/Hematology*.

[3] Eom B. W., Kim Y.-W., Nam B.-H. (2016). The Korean gastric cancer cohort study: study protocol and brief results of a large-scale prospective cohort study. *Journal of Gastric Cancer*.

[4] Abrahao-Machado L. F., Scapulatempo-Neto C. (2016). HER2 testing in gastric cancer: an update. *World Journal of Gastroenterology*.

[5] Bang Y.-J., Van Cutsem E., Feyereislova A. (2010). Trastuzumab in combination with chemotherapy versus chemotherapy alone for treatment of HER2-positive advanced gastric or gastro-oesophageal junction cancer (ToGA): a phase 3, open-label, randomised controlled trial. *The Lancet*.

[6] Hacker U. T., Escalona-Espinosa L., Consalvo N. (2016). Evaluation of angiopoietin-2 as a biomarker in gastric cancer: results from the randomised phase III AVAGAST trial. *British Journal of Cancer*.

[7] Cunningham D., Stenning S. P., Smyth E. C. (2017). Peri-operative chemotherapy with or without bevacizumab in operable oesophagogastric adenocarcinoma (UK Medical Research Council ST03): primary analysis results of a multicentre, open-label, randomised phase 2-3 trial. *The Lancet Oncology*.

[8] Wang G., Hu N., Yang H. H. (2013). Comparison of global gene expression of gastric cardia and noncardia cancers from a high-risk population in China. *PLoS One*.

[9] Ooi C. H., Ivanova T., Wu J. (2009). Oncogenic pathway combinations predict clinical prognosis in gastric cancer. *PLoS Genetics*.

[10] Kim D., Langmead B., Salzberg S. L. (2015). HISAT: a fast spliced aligner with low memory requirements. *Nature Methods*.

[11] Pertea M., Pertea G. M., Antonescu C. M., Chang T.-C., Mendell J. T., Salzberg S. L. (2015). StringTie enables improved reconstruction of a transcriptome from RNA-seq reads. *Nature Biotechnology*.

[12] Love M. I., Huber W., Anders S. (2014). Moderated estimation of fold change and dispersion for RNA-seq data with DESeq2. *Genome Biology*.

[13] Kanehisa M., Araki M., Goto S. (2007). KEGG for linking genomes to life and the environment. *Nucleic Acids Research*.

[14] Yu G., Wang L.-G., Han Y., He Q.-Y. (2012). clusterProfiler: an R package for comparing biological themes among gene clusters. *Omics: A Journal of Integrative Biology*.

[15] Tang Z., Li C., Kang B., Gao G., Li C., Zhang Z. (2017). GEPIA: a web server for cancer and normal gene expression profiling and interactive analyses. *Nucleic Acids Research*.

[16] Rochman Y., Spolski R., Leonard W. J. (2009). New insights into the regulation of T cells by *γ*_c_ family cytokines. *Nature Reviews Immunology*.

[17] Essner R., Huynh Y., Nguyen T., Rose D. M., Kojima M., Hoon D. S. (2001). Functional interleukin-4 receptor and interleukin-2 receptor common gamma chain in human gastric carcinoma: a possible mechanism for cytokine-based therapy. *Journal of Gastrointestinal Surgery*.

[18] Mengus C., Le Magnen C., Trella E. (2011). Elevated levels of circulating IL-7 and IL-15 in patients with early stage prostate cancer. *Journal of Translational Medicine*.

[19] Vainchenker W., Constantinescu S. N. (2013). JAK/STAT signaling in hematological malignancies. *Oncogene*.

[20] Yamada M., Ichikawa T., Ii M. (2005). Discovery of novel and potent small-molecule inhibitors of NO and cytokine production as antisepsis agents: synthesis and biological activity of alkyl 6-(*N*-substituted sulfamoyl)cyclohex-1-ene-1-carboxylate. *Journal of Medicinal Chemistry*.

[21] Kashani B., Zandi Z., Karimzadeh M. R., Bashash D., Nasrollahzadeh A., Ghaffari S. H. (2019). Blockade of TLR4 using TAK-242 (resatorvid) enhances anti-cancer effects of chemotherapeutic agents: a novel synergistic approach for breast and ovarian cancers. *Immunologic Research*.

[22] Everatt R., Tamosiunas A., Kuzmickiene I. (2012). Alcohol consumption and risk of gastric cancer: a cohort study of men in Kaunas, Lithuania, with up to 30 years follow-up. *BMC Cancer*.

[23] Tramacere I., Negri E., Pelucchi C. (2011). A meta-analysis on alcohol drinking and gastric cancer risk. *Annals of Oncology*.

[24] Ferro A., Morais S., Rota M. (2018). Alcohol intake and gastric cancer: meta-analyses of published data versus individual participant data pooled analyses (StoP project). *Cancer Epidemiology*.

