# Supplementary material for: High Expression of Interleukin-2 Receptor Subunit Gamma Reveals Poor Prognosis in Human Gastric Cancer
Source: J Oncol. 2021 Jan 21;2021:6670834. doi: 10.1155/2021/6670834 (PMC7843183; doi:10.1155/2021/6670834)
Supplement: Supplementary Materials — Table S1: characteristics of the patients diagnosed with gastric cancer. Table S2: information of raw sequencing reads and mapping. Figure S1: sequencing samples comparison based on the expression levels of transcripts per million (TPM). N, normal gastric tissues. T, tumor. F, female. M, male. Figure S2: cytokine-cytokine receptor interaction (CCRI) pathway map based on the DEGs between GC and adjacent non-GC gastric tissues. Figure S3: the regulation network of TF-DEG and miRNA-DEG.A. Figure S4: molecular docking results of resatorvid and IL2RG. A. The files named deg_N_T_tpm.txt, deg_NF_TF_tpm.txt, and deg_NM_TM_tpm.txt contain the DEGs and related statistical information. [file 6670834.f1.zip › 6670834.f1/supplementary material-R1.DOCX]

**Supplementary material (ID 6670834)**

**High expression of interleukin 2 receptor subunit gamma reveals poor prognosis in** **human gastric cancer**

**Running title:** Wang et al: Role of IL2RG in human gastric cancer

De-Ping Wang^1*^, Rong Zhao^1*^, Yue-Hong Qi^1,2^, Jing Shen^1^, Jia-Yi Hou^1^, Mei-Yue Wang^1^, Xiao-Gang Bi^3^, Xiao-Qing Guo^4^, Ji-Min Cao^1#^

1 Key Laboratory of Cellular Physiology at Shanxi Medical University, Ministry of Education, and the Department of Physiology, Shanxi Medical University, Taiyuan, China.

2 Department of Anesthesiology, Shanxi Provincial People’s Hospital, Taiyuan, China.

3 Department of General Surgery, Shanxi Provincial People’s Hospital, Taiyuan, China.

4 Department of Liver Disease, Taiyuan Third People's Hospital, Taiyuan, China.

*These authors contributed equally to this work.

**^#^Corresponding Author:**

**Ji-Min Cao**

Institution: Key Laboratory of Cellular Physiology at Shanxi Medical University, Ministry of Education, and the Department of Physiology, Shanxi Medical University, Taiyuan, China.

Address: 56 Xin Jian Nan Lu Rd., Taiyuan 030001, People’s Republic of China.

Email: [caojimin@sxmu.edu.cn](mailto:caojimin@sxmu.edu.cn)

**Supplementary Tables**

**Table S1.** Characteristics of the patients diagnosed with gastric cancer

___________________________________________________________________________

Value

___________________________________________________________________________

Age (years) 59.2 ± 10.4 (mean ± SD, n = 7)

Sex: n (%)   Male 5 (71%), female 2 (29%)

Tumor location and subtype (n) Cardia cancer (1)

Cardia and fundus cancer (1)

Gastric body adenocarcinoma (1)

Curvature and antrum ulcer type adenocarcinoma (1)

Antrum cancer (3)

Pathological grading (n) T4N3M0 (2); T4N2M0 (1);

T4N0M0 (3); T3N0M0 (1)

___________________________________________________________________________________________

**Table S2.** Information of raw sequencing reads and mapping

**_____________________________________________________________________**

Samples Reads Length Bases GC content Mapped reads Mapping rates

___________________________________________________________________________

N-F-1 36079539 100 7215907800 49 35076528 97.22

N-F-2 33974281 100 6794856200 49 33179283 97.66

N-M-1 35041076 100 7008215200 49 33418674 95.37

N-M-2 35162990 100 7032598000 49 34350725 97.69

N-M-3 35119136 100 7023827200 50 34258717 97.55

N-M-4 35097639 100 7019527800 49 34223708 97.51

T-F-1 33522052 100 6704410400 49 32516390 97.00

T-F-2 33777218 100 6755443600 49 32324798 95.70

T-M-1 33755178 100 6751035600 49 32874168 97.39

T-M-2 35200405 100 7040081000 48 34049352 96.73

T-M-3 33934423 100 6786884600 49 32739931 96.48

T-M-4 34977302 100 6995460400 49 33718119 96.40

T-M-5 33247155 100 6649431000 49 32309585 97.18

_____________________________________________________________________

Samples, tissue ID numbers. N, normal. T, tumor. F, female. M, male. Reads, numbers of reads that were sequenced. Length, the length of sequencing reads. Bases, numbers of sequencing bases (sequencing quantity). GC content, GC content of sequencing data. Mapped reads, numbers of reads that were effectively aligned. Mapping rates: the ratio of reads that are effectively aligned.

**Supplementary Figures**


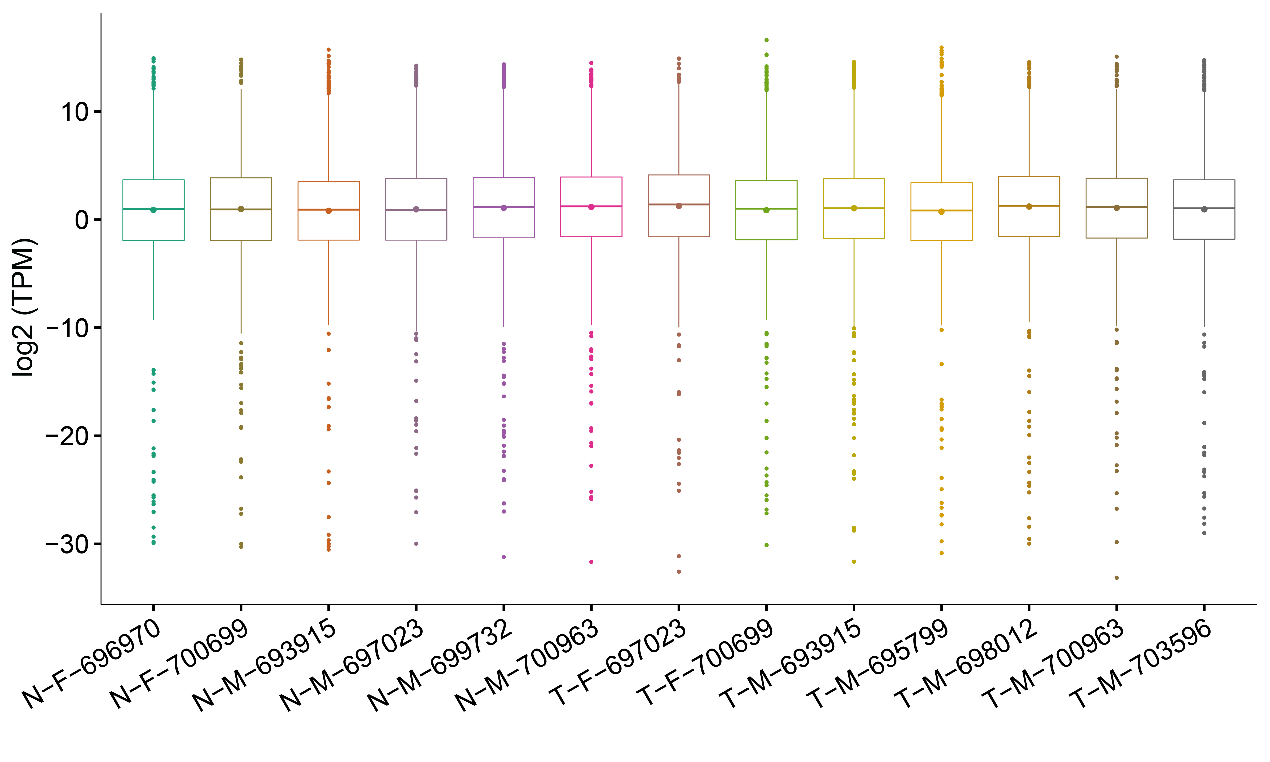


**Figure S1. Sequencing samples comparison based on the expression levels of transcripts per million (TPM).** N, normal gastric tissues. T, tumor. F, female. M, male.


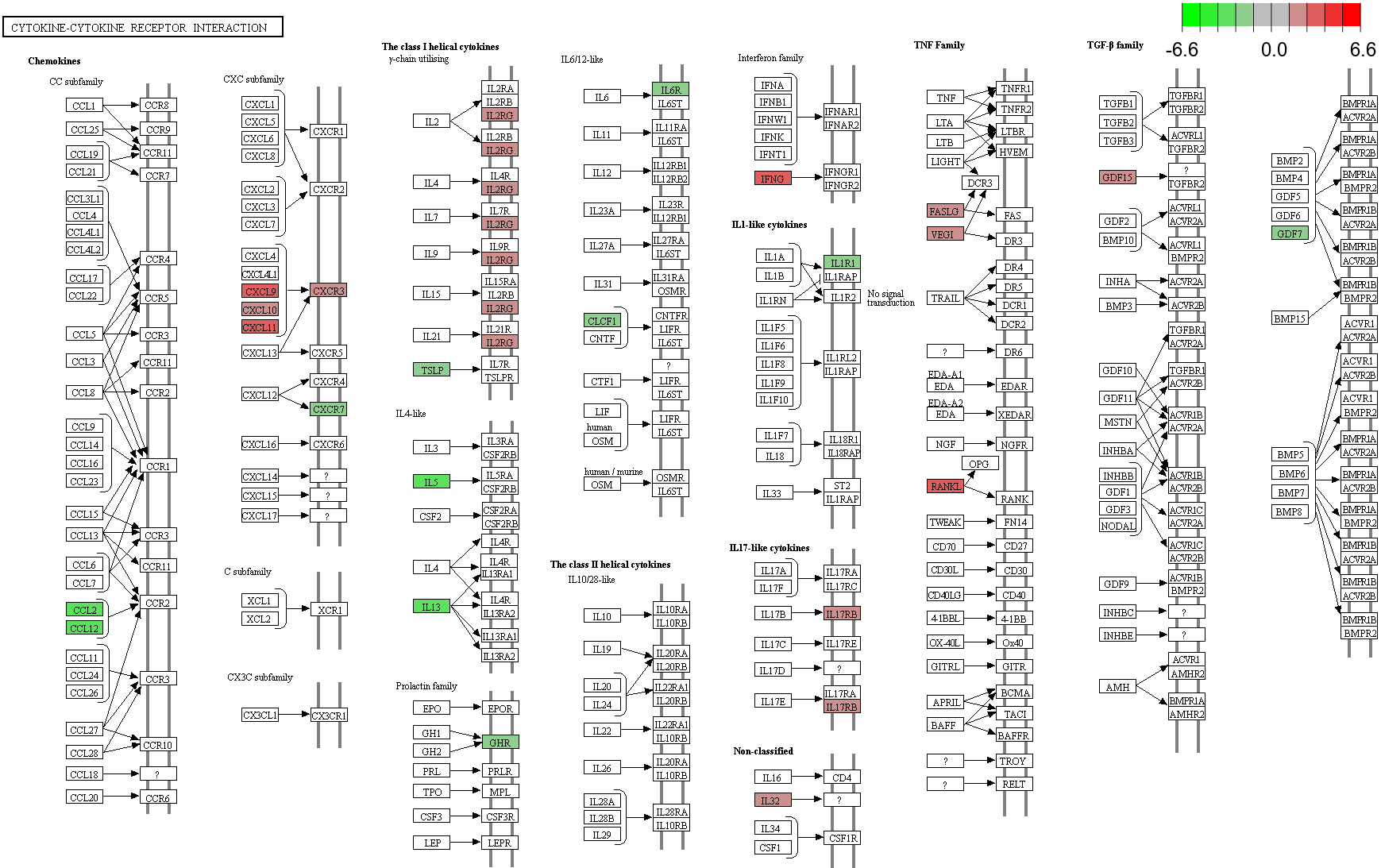


**Figure** **S2. Cytokine-cytokine receptor interaction (CCRI) pathways map based on the DEGs between GC and adjacent non-GC gastric tissues.** Color scale bar in the upper right area of the figure denotes the gene expression level. Genes named in red and purple squares represent the upregulated genes, green squares indicate the downregulated genes. Note that IL2RG gene was enriched in the cytokine-cytokine receptor interaction pathways and was marked purple which indicates upregulated expression.


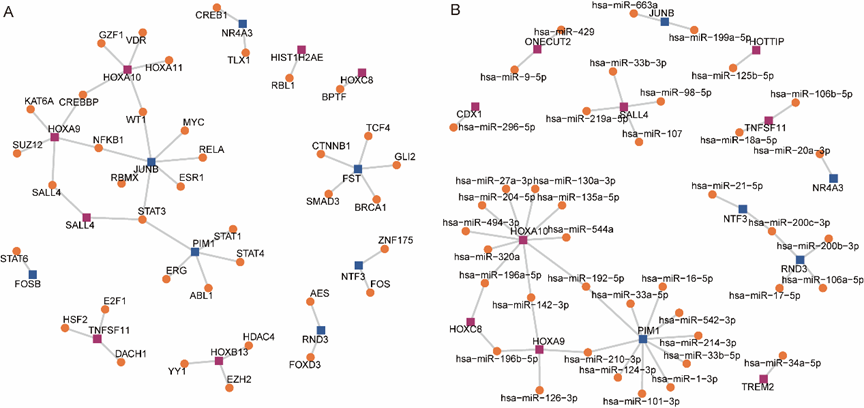


**Figure S3. The regulation network of TF-DEG and miRNA-DEG.** **A,** the regulation network of TF-DEG. Square represents genes; circle represents TF, which was orange to distinguish it from the gene. Red: up-regulated genes; blue: down-regulated genes. **B,** the regulation network of miRNA-DEG. Square represents genes; circle represents miRNA, which was orange to distinguish it from the gene. Red: up-regulated genes; blue: down-regulated genes.


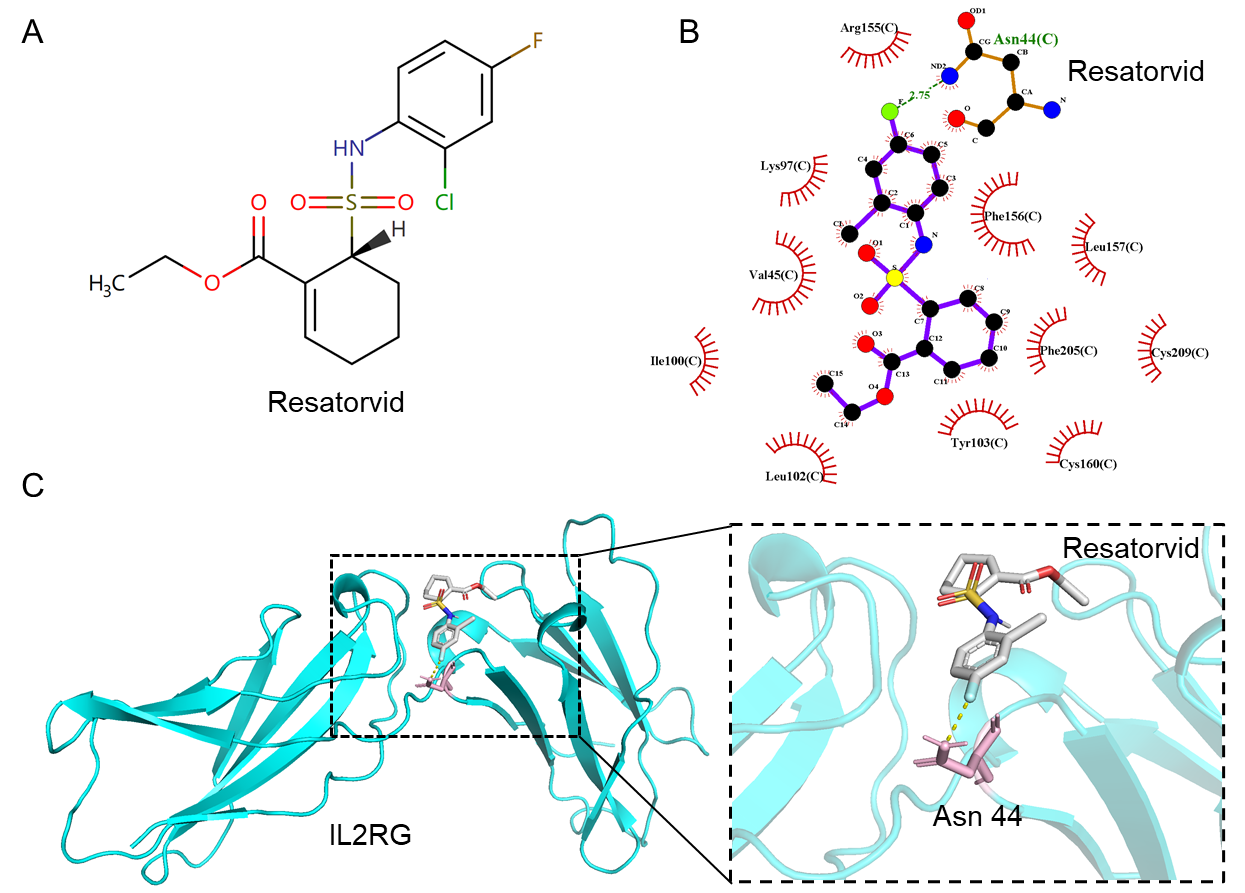


**Figure S4. Molecular docking results of Resatorvid and** **IL2RG. A,** strucuture of Resatorvid. **B,** ligplot results showing the detailed view of the IL2RG-resatorvid interactions, a hydrogen bond is formed between the Asn 44 of IL2RG and the F atom of resatorvid. **C,** the best binding pose of resatorvid with IL2RG (PDB: 5M5E), IL2RG is shown as cartoon, resatorvid as grey sticks, Asn 44 as pink sticks, and hydrogen as yellow dash dot lines.
